# SARS-CoV-2 Transmission Prevention Model Application in a Large Retail Company Before the Vaccine Introduction

**DOI:** 10.3389/fpubh.2022.908690

**Published:** 2022-10-14

**Authors:** Ermanno Vitale, Francesca Vella, Giuliano Indelicato, Andrea Canalella, Salvatore Briguglio, Veronica Pittari, Paola Senia, Denis Vinnikov, Daniele Floresta, Venerando Rapisarda, Veronica Filetti

**Affiliations:** ^1^Department of Clinical and Experimental Medicine, Occupational Medicine, University of Catania, Catania, Italy; ^2^Department of Epidemiology, Biostatistics and Evidence-Based Medicine, Al-Farabi Kazakh National University, Almaty, Kazakhstan; ^3^Health and Safety Manager of Eurospin Sicily and Calabria, Catania, Italy

**Keywords:** COVID-19, SARS-CoV-2, workplace, SARS-CoV-2 prevention, transmission prevention model, telemedicine

## Abstract

On 11 March 2020, following the spread of SARS-CoV-2, WHO declared a pandemic status. The impact on national health and economic systems has been huge. Therefore, many countries took measures to restrict the spread of the virus. Many work activities have been subjected to lockdown measures. However, some production activities, continued to remain open, i.e., large-scale food distribution, food industry, pharmacies, hospitals, etc. In order to contain the spread of the pandemic, public health measures have been implemented by the States to reduce the contagion of the virus in the workplace. Therefore, it was important to implement measures to contrast and contain the spread of SARS-CoV-2/COVID-19 in workplaces. The aim of this study was to adopt and implement a safety protocol useful to restrict the spread of SARS-CoV-2 in a large-scale retail trade company located in the south of Italy, before vaccination, during the first and second pandemic phases also exploiting telemedicine services. Antibody serological test cards were also used during the first pandemic wave and rapid antigenic swabs during the second to detect workers positive for SARS-CoV-2. A population of subjects who worked for another company similar for production activity and distribution on the territory was selected as the control group. During work activities, this group followed the minimum activity protocol provided by the Italian legislation (24 April 2020, Ministry Protocol), which provided the daily monitoring of the body temperature and in the case of SARS- CoV-2 positive subjects the extraordinary sanitation of the workplace. The measures implemented identified the positive subject for SARS-CoV-2 at an early stage. The protocol made it possible to significantly reduce the spread of the virus within large-scale retail distribution, and therefore, to avoid the temporary closure of the stores with a consequent reduction of economic losses compared with the control group.

## Introduction

Severe acute respiratory syndrome coronavirus 2 (SARS-CoV-2) epidemic has brought about profound changes worldwide. Never before has modern society been confronted with a pathogen that has produced so many deaths and huge economic damages (1). To face the devastating health impact due to COVID-19, countries have adopted a number of measures in order to reduce the contagion within the population. Some countries such as China and Italy quickly closed all the main domestic economic activities, favoring smart working (2, 3). Indeed, only essential services were guaranteed (hospitals, pharmacies, supermarkets, food supply chains, petrol stations, the oil industry, etc.). In the second phase of the pandemic, there was a gradual reopening of the previously closed production activities. Large-scale retail trade has been one of the production activities constantly operative during the pandemic, and it even saw an increase in sales due to greater consumption of meals at home (4, 5).

To manage the bulk of users who were continuously going to supermarkets, various procedures were adopted to limit entry into selling points (SPs), in compliance with the national regulations.

The aim of this study was to implement a safety protocol useful to contain the spread of SARS-CoV-2 in a large-scale retail trade company located in the south of Italy, before vaccination, during the first and second pandemic phases also exploiting telemedicine services.

## Materials and Methods

### Study Population

From 1 April 2020 through 31 December 2020, a prospective cohort study was conducted on a population of workers of a large retail company, located in Sicily and Calabria (south of Italy).

The company consisted of 2,117 (100%) workers, 1,987 were distributed across 98 SP (on average 21 workers/SP), and about 130 administrative employees. The tasks performed by the workers were: sales clerk/warehouse worker, and butcher/baker. Almost all the administrative workers continued to work remotely; therefore, they were not included in the study.

Given the purpose of the study, only one inclusion criterion was used: working in the company during the study period.

The study was performed in accordance with the guidelines of the Declaration of Helsinki. Ethical approval was not necessary because all the medical and instrumental examinations were performed according to the Italian law concerning the protection of workers exposed to occupational risks (D. Lgs. 81/2008) (6). All the workers joined the study and informed consent was obtained from all the participants. Employees were interviewed by a trained occupational physician. Medical records, sociodemographic data, information about smoking habits, alcohol consumption, place of residence, and occupational history were collected.

The study period (1 April 2020 to 31 December 2020) was divided as follows: from 1 April through 31 August 2020 was considered to be the first pandemic wave whereas 1 September through 31 December 2020 was the second one.

The study population kept working in the two pandemic phases following a specific safety protocol useful to limit the spread of the SARS-CoV-2, already validated in a previous study (7). Furthermore, a population of subjects who worked for another company similar for production activity and distribution on the territory were selected as the control group. During the working activities, this group followed the minimum activity protocol provided for by the Italian legislation (8), which provided the daily monitoring of the body temperature, and in the case of SARS-CoV-2, positive subjects the extraordinary sanitation of the workplace.

### COVID-19 Risk and Prevention Measures

All the exposed workers were provided with safety shoes, a filter mask for personal respiratory protection (fine particle mask FFP2 in accordance with EU norm EN 95), protective clothing, and gloves. Also, cashier employees were equipped with visors or protective barriers in plexiglass.

Each SP has a different entrance and exit for customers. At the entrance of each SP, there was a 70% hydroalcoholic solution dispenser.

The sales clerk/warehouse worker operated indoors managing cash register activities and the layout of shelves, whereas outdoors he/she took care of the warehouse management.

The activity of the butcher/baker workers was carried out behind the counter with little physical interaction with the customers except for the act of delivering the package.

According to the Occupational Safety and Health Administration (OSHA) and National Institute for Insurance Against Injuries (INAILs) risk stratification, COVID-19 risk was rated as *medium* (SCALE from *low risk* to *very high risk*). Medium exposure risk jobs include those that require frequent and/or close contact with (i.e., within 6 feet) from other people who may be infected with SARS-CoV-2. All of the aforementioned conditions were similar in both study and control group subjects.

In the study group, in order to safeguard the health and safety of workers, according to the guidelines of the Italian Ministry of Health and also to those of the latest scientific literature and major international agencies such as ECDC, OSHA, INAIL, ISS, etc.,…, a safety operating protocol was established to access to the workplace (7).

In summary, in order to implement all the measures to enter the workplace safely, it was necessary to inform each worker about COVID-19–related risks. Each worker signed for acceptance after being informed.

The main information reported was: (a) stay at home in the presence of fever (over 37.5°C) or other flu-like symptoms; (b) not go to and/or stay in the workplace after entry and promptly declare if dangerous conditions such as flu-like symptoms, close contact to a patient with confirmed COVID-19 in the previous 14 days occur; (c) respect all the provisions of the authorities and the employer on entering a workplace. In particular, keep a safe distance, observe the rules of hand hygiene and behave correctly in terms of hygiene; (d) inform the employer of the presence of any flu symptoms during work activities, be careful to stay at an adequate distance from the people around.

The access to the workplace always took place with a mask correctly worn. At the workplace entrance, a worker designated by the employer, with a privacy guarantee, took the body temperature by means of a special contact-less thermometer, pointing it directly at the worker's forehead. In case of detection of a temperature lower than 37.5°C, the worker continued the screening autonomously, through self-detection of oxygen saturation, using a pulse oximeter, after washing hands and sanitizing the index finger with cotton and alcohol; a symptom informative sheet was visibly placed at the entrance (see Table 1).

**Table 1:** Checklist to screen workers before entering the workplace.

**If your values are in this column YOU CAN ENTER**	**If your values are in this column YOU CANNOT ENTER**
Body temperature ≤ 37.5°C	Body temperature > 37.5°C
Oxygen saturation > 95	Oxygen saturation ≤ 95
You are not having difficulty in breathing	You are having difficulty in breathing
You do not have a cough	You have a cough (excluding allergy)
You do not have a fever	You have a fever
You did not have a fever yesterday	You had a fever yesterday
You do not have diarrhea	You have diarrhea
You do not feel nauseous	You feel nauseous
You do not have vomiting	You have vomiting
You have no alterations in the perception of smells	You have alterations in the perception of smells
You do not have altered taste perception	You have altered taste perception
You have no widespread muscle pain	You have widespread muscle pain
You have no eye tearing and redness	You have eye tearing/redness (excluding allergies)
You have no nasal congestion and/or runny nose	You have nasal congestion and/or runny nose (excluding allergies)

**Figure 1:**
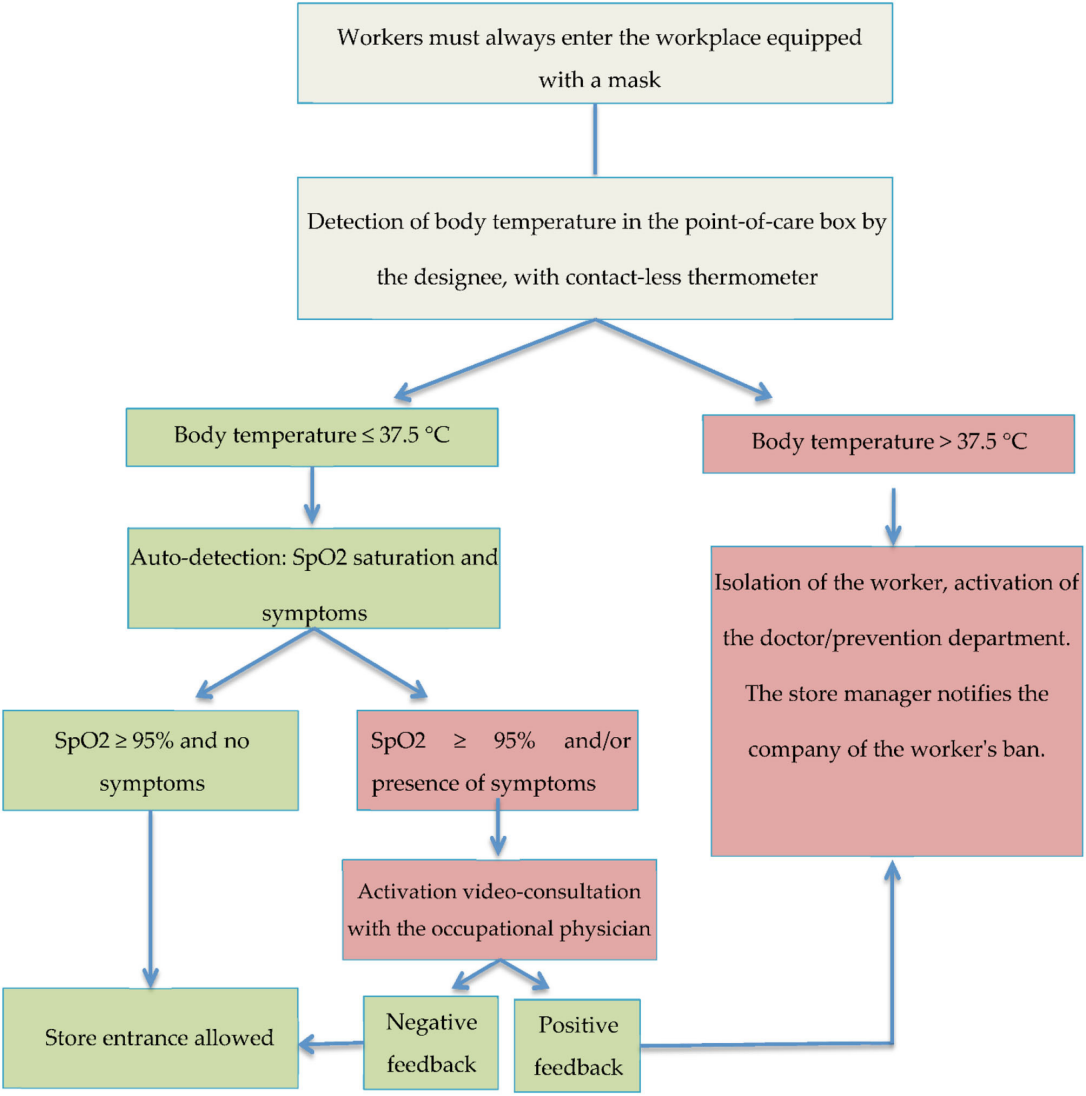
Flow chart with anti-COVID-19 procedures to be followed when entering the company.

If oxygen saturation was below 95% and/or positive symptoms were present, the worker activated telemedicine services with the occupational physician of the Company through a video consultation procedure. On the basis of what the doctor found, the worker could have access to the workplace or return home, in order to contact the family doctor by phone, activating the local health authority. Figure 1 reports the procedure to enter the workplace.

Furthermore, study group workers were checked after the spotting of a positive case for SARS-CoV-2. In particular, from 1 April 2020 through 31 August 2020 (the first pandemic wave) the prevalence of SARS-CoV-2 antibody was assessed in the workplace (IN) by qualitative analysis using the COVID-19 IgG/IgM Rapid Test Cassette. This is a rapid chromatographic immunoassay for the qualitative detection of IgG and IgM antibodies against SARS-CoV-2 in human blood (Lumiratek, SD BIOSENSOR, South Korea) (9, 10). Serological tests were performed after spotting a positive case in order to detect the presence of other asymptomatic cases or with few symptoms. The workers were not quarantined, since all were equipped with suitable PPE in accordance with the Italian legislation (11).

Each worker was tested during the first pandemic wave after coming in contact with a positive case for SARS-CoV-2. Tests were performed at the point-of-care by occupational physicians; and the antibodies were tested in whole blood. Specifically, the test was performed immediately after sample collection: two drops of whole blood (20 μl) followed by two drops (~100 μl) of sample diluent were added to the test well. The test results were read and recorded by the physicians after 10 min. The test was considered positive when the IgM and/or IgG band was/were positive. When no control line appeared or if there was difficulty in interpreting the results, the test was immediately repeated. The test was performed by a physician.

From 1 September through 31 December 2020 (second pandemic wave), rapid antigenic tests were done in the workplace (IN) (12–14). This is an *in-vitro* diagnostic rapid test for the qualitative detection of SARS-CoV-2 antigen (Ag) in human nasal swab specimens from individuals who meet COVID-19 clinical and/or epidemiological criteria. This Ag Rapid Test Device contains a membrane strip, which is pre-coated with the immobilized anti-SARS-CoV-2 antibody on the test line and mouse monoclonal anti-chicken IgY on the control line. Two types of conjugates (human IgG specific to SARS-CoV-2 Ag gold conjugate—binds to the nucleocapsid protein—and chicken IgY gold conjugate) move upward on the membrane chromatographically and react with anti-SARS-CoV-2 antibody and pre-coated mouse monoclonal anti-chicken IgY, respectively. For a positive result, human IgG specific to SARS-CoV-2 Ag gold conjugate and anti-SARS-CoV-2 antibody will form a test line in the result window. Neither the test line nor the control line was visible in the result window prior to applying the patient specimen. A visible control line is required to indicate that a test result is valid.

The tests were carried out on all the workers of a single store, whenever a positive case was reported among the workers of the supermarket.

Control group workers were only checked for body temperature before access to the workplace, as required by the current Italian legislation (8). The control group did not carry out COVID tests in the workplace. The COVID tests carried outside (OUT) were registered by the company, but there was no company planning.

When 3 or more workers were tested COVID positive in the SP, local health authorities ordered the SP's closure.

### Statistical Analysis

Statistical analysis was carried out using the IBM SPSS Statistics 22.0 software. Normality was checked with the Kolmogorov–Smirnov test. The results were reported as the mean and SD or as frequency and percentage. Multivariate logistic regression was used to explore the relations. Student's *t*-test (t) and chi-square (χ^2^) were used to compare means and frequencies, respectively. A *p*-value of <0.05 was considered statistically significant.

## Results

The sample consisted of 1,987 (100%) workers; of these, 64% (*n* = 1,272) were men and 37% (*n* = 735) were women, mean age 41.2 ± 9.8 years, body mass index (BMI) 26.1 ± 6.1, 31% (*n* = 616) were smokers, 13% (*n* = 477) drank at least 1 glass of alcoholic beverage per day. Table 2 reports the main characteristics of the sample.

**Table 2:** Main sample characteristics.

	**Study group**	**Control group**	***p*-values**
	**1,987 (100%)**	**1,798 (100%)**	**n.s**.
Male	1,272 (64%)	1,132 (63%)	n.s.
Age (years)	41.2 ± 11.1	42.1 ± 5.4	n.s.
Years of employment	13.5 ± 8.1	13.9 ± 7.1	n.s.
Smokers (>100 cigarettes/yrs)	616 (33%)	719 (40%)	<0.05
Alcohol consumption (1 drink/last 30 day)	258 (13%)	198 (11%)	n.s
Physical risks: MMC, Sb, Pi	1,987 (100%)	1,798 (100%)	n.s
Salesclerk/warehouse worker	1,490 (75%)	1,317 (73%)	n.s.
Butcher/baker	497 (25%)	481 (27%)	n.s.

*MMC, manual handling of loads; Sb, biomechanical overload of the upper limbs; Pi, incongruous postures; n.s., not significant. Student's t-test (t) and Chi-square (χ^2^) were used to compare means and frequencies, respectively*.

Out of 1,987 (100%) subjects, 1,490 (75%) were salesclerk/warehouse workers, 497 (27%) butcher/baker ones. Occupational health and safety risks for those who worked as salesclerk/warehouse and butcher/baker were: manual handling of loads (MMC), biomechanical overload of the upper limbs (SB), and incongruous postures (Pi).

From 1 April 2020 through 31 August 2020 (first pandemic wave), 232 (12%) subjects performed the serological test to assess the SARS-CoV-2 antibody. Of these, 97% (*n* = 224) of workers underwent serologic screening in the workplace (IN) and 3% (*n* = 8) outside the workplace (OUT).

Among the subjects tested IN, 8 (3%) asymptomatic workers were positive for anti-SARS-CoV-2 IgM. In 7 cases of this, the positivity was confirmed by swab rt-PCR. Nobody was positive for anti-SARS-CoV-2 IgG.

Among the subjects tested OUT, 1 (1%) was found positive for anti-SARS-CoV-2 IgM; the positivity was confirmed also by the rt-PCR test. Nobody was found positive for anti-SARS-CoV-2 IgG.

From 1 September through 31 December 2020 (second pandemic wave), serological tests were no longer performed, instead favoring the use of nasopharyngeal swabs. Overall, 1,289 (100%) nasopharyngeal swab tests were carried out, 1,236 (96%) in the workplace (IN) and 53 (4%) outside the workplace (OUT). On the total of tests carried out IN, 61 workers were found positive (5%), instead, among tests carried outside the OUT, 31 (58%) turned out as positive.

In the same periods, the control group (*n* = 1,798) during the first pandemic wave carried out 35 (2%) serological OUT tests of which 5 (16%) were positive for SARS-CoV-2 IgM and 1 (3%) for IgG. From 1 September through 31 December 2020, 118 (7%) OUT tests were carried out, of which 48 (41%) were positive. Table 3 reports the results of the screening carried out during the two pandemic waves (I and II) and the results of the control group.

**Table 3:** Results of the tests carried out between the study group and the control group during the first and second pandemic waves.

	**Test conducted**	**Study group**	**Control group**	***p*-values**
		**1,987 (100%)**	**1,798 (100%)**	
First pandemic wave	Serological test (IN)	224 (11%)	0	n.c.
	IgM Positive	8 (4%)	0	n.c.
	IgG Positive	0	0	n.c.
	Serological test (OUT)	8 (1%)	35 (2%)	<0.05
	IgM Positive	1 (13%)	5 (16%)	n.s.
	IgG Positive	0	1 (3%)	n.s
	Total serological tests conducted (IN e OUT)	232 (12%)	35 (2%)	<0.05
	IgM Positive	9 (4%)	5 (16%)	<0.05
	IgG Positive	0	1 (3%)	n.c
Second pandemic wave	Positive to rt-PCR	7 (1%)	1 (1%)	
	Swab tests (IN)	1,236 (62%)	0	n.c.
	Positive	61 (5%)	0	n.c.
	Swab tests (OUT)	53 (4%)	118 (7%)	<0.05
	Positive	31 (58%)	48 (41%)	<0.05
	Total swab tests conducted (IN e OUT)	1,289 (65%)	118 (7%)	<0.05
	Total positive Swab tests	92 (7%)	48 (41%)	<0.05

*n.s., not significant; n.c., not calculable; IN, in workplace; OUT, outside workplace. Chi-square (χ^2^) was used to compare the frequencies*.

Since the introduction of the protocol (April 2020), 105 (5%) workers of the study group were blocked from entering the SP on account of the presence of one or more of these symptoms: fever (52%), cough (41%), loss of taste and smell (22%), muscle or joint pain (11%), nausea or vomit (11%), oxygen saturation <95% (7%). Of these 105 workers, 20% (*n* = 21) turned out positive for COVID-19 rt-PCR. The main symptoms of 21 subjects were: 7 (33%) had fever, 5 (24%) loss of taste and smell, 4 (19%) cough, 3 (14%) cough and loss of smell, and 2 (10%) vomit. In the control group, 7 (1%) workers were not allowed in the SP; of these, 2 (29%) were positive for COVID-19 rt-PCR swab (see Table 4).

**Table 4:** Workers blocked on entering SP.

**Pandemic period**		**Study group**	**Control group**	***p*-values**
		**1,987 (100%)**	**1,798 (100%)**	
First wave	Blocked	61 (3%)	(1%)	n.c.
	rt-PCR Positive	5 (8%)	0	n.c.
Second wave	Blocked	44 (2%)	(1%)	<0.05
	rt-PCR Positive	17 (38%)	2 (40%)	n.c.
Total	Blocked	105 (5%)	7 (1%)	<0.05
	rt-PCR Positive	21 (20%)	2 (29%)	n.c.

*n.c., not calculable. Chi-square (χ^2^) was used to compare the frequencies*.

Since the introduction of the experimental protocol on 98 (100%) SPs, 1 (1%) was temporarily closed by the local health authorities due to the presence of more than 2 cases (*n* ≥ 3). While out of 79 (100%) SPs of the control company, 6 (8%) were closed.

## Discussion

The role played by large-scale distribution companies in ensuring the safety of workers and users during the pandemic waves has revealed itself as dramatically important. In fact, during the study period, INAIL data recorded only 0.03% of workplace accidents from COVID contagion in large-scale distribution (15). In this survey, the study sample and the control group were homogeneous for all the anthropometric parameters, with a statistically significant difference in the terms of smoking. This is possibly because of a previous protocol against smoking habits that the company had previously implemented (16).

The anti-COVID protocol applied to the company saw a different output in the two pandemic phases. In the first pandemic wave, the implementation of a company screening with rapid serological tests on a card made it possible to identify seven positive subjects against one in the control group where no tests were carried out within the SP. However, although the difference in positives detected with a rapid serological test between the two groups was not statistically significant, the absolute value of the positives in the study group was 7 times greater than the control group.

The low number of positive subjects is explained by the low number of cases in the regions of Sicily and Calabria in the first pandemic wave (17).

However, in a study conducted by Gresh et al. (18) on 2,241 subjects, tests performed using the LumiraDx SARS-CoV-2 Ag showed a low false-negative rate of 3.8%. This highlights how useful this type of test was as a screening tool. In fact, other authors also agree on the usefulness of the serological test as a screening tool for subjects with symptoms and close contact (19).

In the second wave of the pandemic, with the spread of rapid antigenic tests, the identification of positive cases was more immediate. In fact, while the rapid serological test took 2/3 weeks from contact with a positive subject to identify an antibody response, whereas with rapid antigenic tests only 5/7 days from contact were needed to spot new cases and prevent the spread of the virus early inside the SP. In fact, with the application of the anti-COVID protocol, it was possible to identify 61 (5%) positive subjects early. Whereas, in the control group, the presence of any positives among the workers emerged every time the worker carried out the test outside the workplace, thus, favoring the possibility of permanence of asymptomatic or paucisymptomatic positive subjects in the SP.

A study by Torres et al. (20) shows that the use of rapid antigenic tests has found strong support in the cases of asymptomatic subjects, with a 100% test specificity and a sensitivity ranging between 35.7 and 50.8% in group two observed, as compared with rt-PCR test results. This characteristic made it possible to identify infected subjects in their still asymptomatic phase.

It was also observed that the number of positives present in the study group was significantly greater than in the control group. This can be explained by the fact that many subjects who did not develop symptoms remained unknown within the control group, potentially continuing to spread the virus in the workplace. In fact, the total number of subjects in the study group who resulted positive out of the total 1,289 tests was 92 (7%), while out of the total 118 tests, 48 (41%) turned out as positive in the control group.

It was also decided to adopt this protocol since the studies published at that time already showed that fever did not occur in all the cases (21, 22). In fact, clinical data suggested that only in 43.8% of cases did fever occur in the early stages of the SARS-CoV-2 infection (22), and in the other cases, other symptoms were present, such as sputum (15.4–33.7%) (21–24) dyspnoea (18.7–31%) (22, 23), pharyngodynia (5–17.4%) (21–23), nasal congestion (4.8%) (22), dizziness (9.4%) (21), and diarrhea (2–10.1%) (21–24).

Subsequently, a meta-analysis showed that the main clinical symptoms in COVID-19 patients were fever (88.5%), cough (68.6%), myalgia or fatigue (35.8%), expectoration (28.2%), and dyspnoea (21.9%) (25). Minor symptoms included headache or dizziness (12.1%), diarrhea (4.8%), nausea, and vomiting (3.9%) (25). Other studies showed that 85.6 and 88.0% of patients reported olfactory and gustatory dysfunctions, respectively (26).

Clearly, the symptoms described referred to those caused by the variants of the moment. As is known, the new variants of the virus are causing different symptoms than the previous ones, symptoms linked to the upper respiratory tract being the most common (27, 28).

The application of the anti-COVID protocol prevented access to work for 21 (20%) positive subjects, who otherwise might have spread the virus. In fact, only 4% (*n* = 4) of the subjects blocked at entry would have been blocked even without the company protocol being applied, as they manifested a fever.

The application of the protocol also allowed the explosion of the positive outbreak and the consequent temporary closure of the SP in only 1% (*n* = 1) out of 98 (100%) SPs of the study group against a temporary closure of the 8% (*n* = 6) of SPs which did not apply the protocol. This has in fact resulted in a lower economic impact in terms of losses linked to the days of closure.

The main weaknesses of the study were: it was not possible to have an accurate reporting of the total number of tests performed by the control group workers. In fact, some workers who did an OUT test with a negative result may have not communicated its result to the company later on. Furthermore, during the first pandemic phase in Sicily, the spread of infections was low, the maximum spread of the virus being in July and August, with more than 50 cases per day (17).

The strengths of the study were the high sample size and the long period of observation (about 9 months).

The cost and budget impact of the intervention were low. In particular, the total cost of the kits and medical performances was around €37,000. Considering the company's 2020 turnover (over €700 million), the impact on the company balance sheet considering the risk/benefit ratio well-justified the implementation of the protocol.

## Conclusion

The protocol allowed to identify the workers with symptoms suggestive of COVID-19 at an early stage, also through the implementation of telemedicine services, therefore, preventing them from entering the SP and potentially spreading the virus to other fellow workers.

Performing rapid tests inside the company made it possible to identify and isolate 11 workers who otherwise would have continued to work and be a potential source of contagion.

The implementation of these measures, therefore, had important health implications, as it contributed to reducing the spread, as well as an economic impact, because it avoided potential COVID-19-related absences and also prevented the SPs from being shut down when positive cases occurred (*n* ≥ 3).

Avoiding the closure of the SP for days certainly produced benefits in terms of expenditure as the average revenue per SP amounts to about €45,000/day.

Preventing contagion and therefore the absence of workers for days also allowed the SP to keep the work organization almost unchanged, without having to resort to double shifts or transfer of workers from other locations.

The development of an anti-COVID effectiveness protocol is important above all in light of the unpredictability of the mutations that the virus can manifest, therefore, requiring again the adoption of important measures to fight the pandemic.

## Data Availability Statement

The raw data supporting the conclusions of this article will be made available by the authors, without undue reservation.

## Ethics Statement

Ethical review and approval was not required for the study on human participants in accordance with the local legislation and institutional requirements. The patients/participants provided their written informed consent to participate in this study.

## Author Contributions

EV, DF, and VR contributed to designing and implementing this study and to the writing of the manuscript. FV, GI, AC, SB, VP, PS, DV, VR, and VF supervised the study. All the authors contributed to the article and approved the submitted version.

## Conflict of Interest

DF was employed by Eurospin. The remaining authors declare that the research was conducted in the absence of any commercial or financial relationships that could be construed as a potential conflict of interest.

## Publisher's Note

All claims expressed in this article are solely those of the authors and do not necessarily represent those of their affiliated organizations, or those of the publisher, the editors and the reviewers. Any product that may be evaluated in this article, or claim that may be made by its manufacturer, is not guaranteed or endorsed by the publisher.
